# Trauma-focused cognitive behavioral therapy with unaccompanied refugee minors: a case series

**DOI:** 10.1186/s12888-015-0645-0

**Published:** 2015-10-23

**Authors:** Johanna Unterhitzenberger, Rima Eberle-Sejari, Miriam Rassenhofer, Thorsten Sukale, Rita Rosner, Lutz Goldbeck

**Affiliations:** Catholic University Eichstätt-Ingolstadt, Clinical and Biological Psychology, Ostenstrasse 25, 85071 Eichstätt, Germany; University Ulm, Clinic for Child and Adolescent Psychiatry, Steinhövelstrasse 5, 89075 Ulm, Germany

**Keywords:** Refugee, Adolescent, Unaccompanied, Treatment, Trauma-focused, PTSD

## Abstract

**Background:**

Unaccompanied refugee minors (URMs) are a group who are vulnerable to developing posttraumatic stress symptoms (PTSS). However, they rarely receive the treatment that is indicated and there are no treatment studies focusing exclusively on this group of adolescents. This case study evaluates the feasibility of trauma-focused cognitive behavioral therapy (TF-CBT) for URMs with PTSS.

**Method:**

A health care utilization sample of *N* = 6 was assessed prior to and after treatment with TF-CBT. Therapists were asked to report differences in treatment application and content in comparison to TF-CBT standard protocol.

**Results:**

We found moderate to high levels of PTSS at baseline and a clinically significant decrease in symptoms at posttest. Some modifications to the TF-CBT protocol were made with regard to affective modulation which required more sessions than usual whereas fewer caregiver sessions were conducted.

**Conclusion:**

TF-CBT is feasible in reducing PTSS in severely traumatized URMs. Further research with controlled trials is necessary.

**Trial registration:**

The trial registration: ClinicalTrials.gov Identifier NCT01516827. Registered 13 December 2011.

## Background

Asylum applications in industrialized countries increased 28 % in 2013 compared to 2012 [1]. Worldwide 51.2 million people are in flight, while 16.7 million actually cross borders to live in another country. Young people under 18 make up about 50 % of this population. Unaccompanied refugee minors (URMs) are defined as children and adolescents “who are separated from both parents and are not being cared for by an adult who, by law or custom, is responsible to do so” [2].

According to the growing volume of literature there is evidence that young refugees in general [3, 4] and URMs in particular [5–7] are confronted with many traumatic experiences in their home countries and during flight. Several studies indicate high rates of posttraumatic stress disorder (PTSD), depression or anxiety disorders in young refugees [8–12]. In addition, late-onset PTSD, a phenomenon which develops after early symptoms of depression and anxiety [13] is easily overlooked and pushes prevalence rates even higher. Independently from the time of incidence, there seems to be a high risk of symptoms and diagnoses becoming chronic [8, 14].

For various reasons, URMs are seen as an especially vulnerable group within the refugee population. The dose effect proposed by Mollica and colleagues [15, 16] assumes that posttraumatic stress can be predicted from the number of traumatic experiences. In comparison to adolescent non-refugees and accompanied refugees, URMs are reported to have experienced a higher number of traumatic/stressful events [5, 17–20]. Furthermore, the traumatic experience of separation from a significant person (e.g., parents) is associated with an increased risk of PTSD [18, 21]. In addition, the family is seen as “a buffer against stress” [3] in the context of migration which is, by definition, lacking for URMs. Therefore, Huemer et al. [22] conclude that “URMs are a highly vulnerable group who likely suffers from more psychiatric morbidity than comparable populations”. This conclusion is confirmed by comparison studies revealing that URMs manifest greater prevalence or severity of psychopathology than adolescent non-refugees and accompanied refugees [5, 17, 18, 20, 22, 23].

The number of URMs affected by traumatic stress symptoms or PTSD in industrialized countries ranges from 20 to 53 % [7, 13, 18, 24, 25] and remains stable over time [7, 26, 27]. Furthermore, Smid and colleagues [13] observed 18 % of late-onset PTSD in their URM sample two years after their arrival in the Netherlands. A German study revealed a prevalence of 19 % for PTSD in accompanied young refugees with only one of the affected children and adolescents receiving mental health treatment [12]. Bean, Eurelings-Bontekoe, Mooijart, and Spinhoven [28] reported a rate of 49 % for unmet psychosocial support need in URMs in the Netherlands. Hence, the question arises how URMs can gain access to effective mental health care and which treatment approach is most feasible for this distressed population of young patients from divergent cultural backgrounds.

In a recent review, trauma-focused cognitive behavioral therapy [29] was identified as being one of the most successful treatment protocols for trauma-related symptoms in children and adolescents [30]. However, this review did not focus on refugee children. Tyrer and Fazel [31] examined treatments for young accompanied refugees and found nine studies reporting significantly decreased posttraumatic stress symptoms (PTSS): four using cognitive behavioral therapy (CBT) or trauma-focused CBT (TF-CBT), three using narrative exposure therapy (NET) or NET for children (KIDNET), two using creative, and one using systemic approaches. Eberle-Sejari, Nocon, and Rosner [32] conclude that although studies evaluating PTSD treatments for young refugees are scarce and those published suffer from methodological problems there is some evidence that a specific trauma-focused approach can be effective in reducing PTSS in young refugees.

To our knowledge, there is no study explicitly investigating PTSD treatment for URMs. TF-CBT is the best supported therapy for traumatized young people at the moment with efficacy trials from different industrialized countries [33–35], with proven feasibility for refugee children [36, 37], and in different international settings like Cambodia and Zambia [38–41]. It, therefore, seems to be a promising approach for the group of URMs suffering from PTSS. The advantages of this treatment for refugee youth are, for instance, it is “skills-based (…), theoretically grounded (…), time-limited (…), and opening many opportunities for cross-cultural modifications while maintaining fidelity” [36]. Furthermore, adolescents with complex PTSD may benefit from TF-CBT [42], a phenomenon to be found in URMs with multiple traumatic experiences.

The feasibility of TF-CBT and its effectiveness in reducing PTSS in URMs is being tested in this case series. Quantitative and qualitative outcome data are provided to answer not only the question about symptom reduction but also about modifications in therapy setting and content where applicable.

## Method

### Participants

The study participants were six adolescents who applied to two trauma outpatient clinics in Germany who had not previously been designated for study participation. Therefore, they represent a selected health care utilization sample. Both outpatient clinics are study centers of a large-scale randomized controlled trial (RCT) to evaluate TF-CBT for children and adolescents with PTSD, *treatchildtrauma* [43] and specialize in mental health care for traumatic stress disorders. The adolescents were either randomized within the RCT study or – if inclusion criteria for the study were not fulfilled (age > 17 years, *n* = 1; involvement of a professional translator, *n* = 2) – treated according to the TF-CBT manual but without study participation. Sufficient knowledge of German was not mandatory outside of the RCT as professional translators were available. The adolescents had to fulfill the following criteria: (1) refugee or asylum seeking, (2) aged between 13 and 18, (3) arrived in Germany without parents or a caregiver, (4) primary diagnosis of PTSD based on the Clinician Administered PTSD Scale for Children and Adolescents (CAPS-CA) [44] or the Posttraumatic Diagnostic Scale (PDS) [45], and (5) an attachment figure (e.g., educator in child and youth welfare (CYW) facility) available to accompany the adolescent to therapy.

### Procedure

Adolescents were referred to the outpatient clinics by their educators, social workers or child and adolescent psychiatrists and were initially invited to a screening appointment. If the screening was positive and age was ≤ 17 years, they (*n* = 3) were assessed by means of the CAPS-CA, the Schedule for Affective Disorders and Schizophrenia for School-Age Children [46] to identify disorders other than PTSD, and a number of questionnaires. For orientation, two subtests of an intelligence scale [47] were administered. Assessments were conducted by trained, blinded psychologists. Non-study participants (*n* = 3) went through the usual diagnostic procedure of the outpatient clinic which is much shorter than the study diagnostic with *n* = 1 CAPS-CA only and *n* = 2 PDS only. In these cases, post ratings were conducted by the therapists themselves. All assessments took place before (baseline, T1) and after treatment (post, T2). The RCT was approved by the ethics review board of the University Ulm. In addition, all participants gave written informed assent and their guardians gave written informed consent for participation.

### Measures

The CAPS-CA [44] was used to obtain PTSD diagnoses according to the Diagnostic and Statistical Manual of Mental Disorders, 4^th^ edition (DSM-IV) and to determine symptom severity. The interviewer rates the frequency and intensity of PTSS during the previous four weeks from *never* (0) to *almost daily* (4) and *none* (0) to *extreme* (4). A symptom is evident if the frequency is rated at least *sometimes* (1) with at least *moderate* intensity (2). The PDS [45] was used to determine PTSD diagnostic status in accordance with DSM-IV criteria and symptom severity; symptoms are rated on a scale from 0 to 3 in the previous four weeks with a maximum of 51and ≥ 21 moderate/ severe, ≥ 36 severe. The PDS was filled in with the help of a clinician (and a translator if necessary) in an interview-like setting. The validity of the German versions of both measures is well reported [48, 49].

Our open-ended questionnaire for therapists asked for a short summary of each of their patients including life-situation, reasons for consultation, treatment aims and treatment content. Furthermore, therapists were asked about modifications to TF-CBT with regard to content, material and setting (“In comparison to other TF-CBT cases, which modifications in the treatment manual were applied?”). Finally they could report barriers and challenges in therapies with URMs compared to therapies with non-refugees (“Did you face specific barriers and challenges in your therapeutic work compared to non-refugee TF-CBT patients? Did you identify specific needs of your patient in comparison to non-refugee TF-CBT patients?)”, if they worked with interpreters and if so, what experiences they had had.

### Treatment

TF-CBT [29] includes the following eight components subsumed within the PRACTICE acronym: psychoeducation and parenting skills, relaxation, affective modulation, cognitive processing, trauma narrative, in vivo exposure, conjoint child/caregiver session, and enhancing safety and future skills. Contents according to the TF-CBT manual are described in Table 1. The components are covered in 12 to 15 90-min parallel sessions with the child and the caregiver. The first four components, usually covered in four sessions, constitute the toolbox encompassing all the skills that prepare the adolescent and the caregiver for the trauma-focused work. About four sessions are scheduled to write (or draw or tape) the trauma narrative which is a graduated exposure in sensu; cognitive processing II, namely about trauma-related cognitions, is included in this component. If necessary, one or two sessions can be used for in vivo exposure with trigger stimuli after the narrative. This is followed by a conjoint session with both the child and the caregiver. The child shares the narrative with the caregiver and both answer each other’s questions. One or two sessions round off the treatment with the focus on future safety and coping.

**Table 1:** Content of weekly TF-CBT double sessions according to Cohen et al. [29]

Session	Topic and description
1	**P**sychoeducation, Parenting skills:
	Psychoeducation about trauma, trauma-related symptoms and the rationale of TF-CBT, normalizing the symptoms, teaching positive parenting skills (e.g., praise, active ignoring, contingency management strategies)
2	**R**elaxation:
	Information about the rationale of relaxation, demonstration and practicing relaxation techniques (progressive muscle relaxation, and/or controlled breathing)
3	**A**ffective modulation:
	Explanation of rationale, identification of feelings, expression of feelings, rating the intensity level of emotions, positive self-instructions, coping with difficult/ unpleasant emotions, thought stopping, teaching problem-solving strategies
4	**C**ognitive processing I:
	Outline the cognitive triangle, identify dysfunctional thoughts in daily life, help the child generate more accurate and helpful thoughts
5-8	**T**rauma narrative:
	Decide on a format for the narrative, describe the perception of event including the worst moment, read the narrative, add thoughts and feelings
9	Cognitive processing II:
	Exploring and correcting cognitive errors concerning the traumatic experience (e.g., cognitions of guilt).
10	**I**n vivo exposure:
	Explaining the rationale, development of a “plan” (if necessary instruct caregiver for co-therapeutic expo)
11	**C**onjoint child/caregiver session:
	Explaining the rationale, prepare child and parent for conjoint session, sharing the trauma narrative, answering questions, increase communication
12	**E**nhancing safety and future skills:
	Developing a feeling of safety, a safety plan, teaching safety skills

*TF-CBT* trauma-focused cognitive behavioral therapy

There were between 12 and 28 treatment sessions, one session lasting approximately 100 min (as the German health care system prescribes 50 min for one session). Within that number of sessions, we set up 3 to 23 sessions for the caregiver (*M* = 9.8). Two therapies were conducted with the help of professional translators (adolescent sessions only). Therapies were conducted by three (two female, J.U., M.R.; one male, T.S.) therapists previously trained in CBT. They all received additional TF-CBT training lasting at least two days. They worked their way through the manual, completed the web-based learning course (http://tfcbt.musc.edu/) and had treated 3 to 15 cases with TF-CBT before. There was biweekly supervision by experienced clinicians (R.R., L.G.). In addition, case-based conference calls were offered twice a month by one of the developers of TF-CBT (Anthony Mannarino) and one experienced TF-CBT trainer (Laura Murray) to ensure manual fidelity. This offer was used regularly in all but one case (No. 3).

### Statistical analysis

As patients were assessed with different measures the sample was divided into two groups, one for CAPS-CA and one for PDS. We computed Wilcoxon signed rank tests for each group to check for PTSS changes. On the individual level clinically meaningful symptom reduction was assessed with the reliable change index [50]. Clinically meaningful change (responder) for the CAPS-CA was set at 15 points [51] and for the PDS at 8 points based on the normative sample [45]. The meeting of diagnostic criteria (DSM-IV) after treatment was determined by examining the fulfilled criteria according to CAPS-CA or PDS. Recovery was defined as not fulfilling diagnostic criteria for PTSD as well as clinically meaningful improvement. In addition, percentage improvements in symptom scores were computed for each case [52].

## Results

### Sample characteristics

Our sample consisted of *N* = 6 URMs, age *range* 16 – 18 (*Md* = 17), two (33.3 %) of whom were female. They arrived in Germany between 12 and 25 months before baseline assessment and most of them had completed basic school education (junior high school; 66.7 %). Asylum status was permanent for one, temporary for one, and tolerated for four of the participants. No changes in asylum status occurred during the course of therapy. The adolescents experienced a *range* of 2 – 6 traumatic events (*Md* = 5; assessed using CAPS-CA or PDS; they all happened prior to the arrival in Germany). Four adolescents had experienced chronic stressors making it difficult to differentiate single experiences. Hence, we assume that the number of traumatic events is higher than the number reported. All participants presented with significant symptom levels at baseline and the majority was most impaired by hyperarousal symptoms. No comorbid disorders were diagnosed. Short case presentations and the summary of treatment goals are given in Table 2.

**Table 2:** Overview of each participant’s case history and treatment aims

No	Brief case history	Treatment aims
1	17-year old girl from Somalia, living in a residential group of a child and youth welfare (CYW) facility, attending a preparatory class for school (did not attend school in Somalia). Parents live in Somalia; they are in contact irregularly. She fled with a family member who died during flight; three brothers died from violence. Presents with sleeping disorder, panic-like fright reaction, impulsive, angry behavior, auto-aggressive behavior.	Reduction of sleeping disorders
		Reduction of self-injurious behavior
		Reduction of states of panic
		Habituation to traumatic experiences
		Correction of cognitive bias
		Enhancing a feeling of safety
2	17-year old male patient from Afghanistan, living in a CYW facility and attending a cooperation class in preparation for vocational school. He has brothers in Germany, his parents also fled. He doesn’t know what happened to them. Presents with massive sleeping disorders, nightmares and (auto-)aggressive behavior.	Reduction of sleeping disorder
		Reduction of (auto-)aggressive behavior
		Reduction of nightmares
		Habituation to traumatic experiences
		Correction of cognitive biases
		Enhancing a feeling of safety
3	17-year old male patient from Afghanistan, living in a CYW facility and attending junior high school. His family was threatened by the Taliban, family members died, flight with many traumatizing experiences. He presents with sleeping disorders (<2 h), severe intrusions, depressed and sad mood.	Reduction of sleeping disorder
		Reduction of intrusions
		Habituation to traumatic experiences
		Correction of cognitive biases (e.g., “I didn’t care enough for my mum”)
		Enhancing a feeling of safety
4	16-year old girl from Iran, living with her two sisters in a CYW facility and attending junior high school. Parents are alive, but not in Germany. She is in contact with mother and siblings. Father threatened to kill family, she was abused during flight. Presents with massive sleeping disorders and severe intrusions.	Reduction of sleeping disorder
		Reduction of intrusions
		Habituation to traumatic experiences
		Enhancing a feeling of safety
5	18-year old male patient from Afghanistan, living in his own apartment that is part of a CYW facility. He completed junior high school during therapy and started an apprenticeship. His parents and siblings are alive; one brother disappeared during flight. He presents with sleeping disorders, nightmares, flashbacks, dissociative symptoms and strong headache.	Reduction of sleeping disorder
		Reduction of dissociative symptoms
		Reduction of flashbacks
		Habituation to traumatic experiences
		Correction of cognitive biases (guilt)
		Enhancing a feeling of safety
6	17-year old male patient from Afghanistan, living in a CYW residential group. He attends a preparatory class for secondary school and did several internships during therapy. He has no contact to his family except for one uncle in Germany. In Afghanistan he lived in the desert and had no previous experience of urban life. His brother was murdered. Presents with massive sleeping disorders (nightmares, sleepwalk, waking up during night), severe intrusions, strong headache, depressive and sad mood.	Reduction of sleeping disorder
		Reduction of intrusions
		Habituation to traumatic experiences
		Correction of cognitive biases (e.g., “I walked out on my family.”)
		Enhancing a feeling of safety

### Quantitative outcome data

At baseline, all patients showed moderate to severe symptom levels according to PTSD measures (CAPS-CA *Md* = 52, *range* 38–61; PDS *Md* = 32, *range* 31–33; see Table 3). At posttest symptom scores were *Md* = 14.5 (*range* 5–20) for the CAPS-CA and *Md* = 12.0 (*range* 10–14) for the PDS. Wilcoxon signed rank tests indicated significantly decreased PTSS in both groups by *W*^−^ = 0 (*p* < .001) and negative rank sums only (*Z*-values are not reported as the sample sizes are too small). The CAPS-CA group improved on average by 73.4 %, the PDS group by 62.5 %.

**Table 3:** Age, sex, country of origin, and individual scores of patients; change of symptom severity in %, median and range of sum scores for sub-samples

No	Age	Sex	Country of origin	Score pre	Score post	Change
				CAPS-CA	CAPS-CA	
1^a^	17	Female	Somalia	50	5	90 %
2^a^	17	Male	Afghanistan	54	17	69 %
3	17	Male	Afghanistan	61	20	67 %
4^a^	16	Female	Iran	38	12	68 %
*Md* (range)	17 (16–17)			52 (38–61)	14.5 (5–20)	
				PDS	PDS	
5	18	Male	Afghanistan	33	14	58 %
6	17	Male	Afghanistan	31	10	68 %
*Md* (range)	17.5 (17–18)			32 (31–32)	12 (10–14)	

*CAPS-CA* clinician administered PTSD scale for children and adolescents, *PDS* posttraumatic diagnostic scale

^a^participated in RCT

On the individual level, percent improvement was > 50 % without exception (see Table 3). Clinically meaningful change was observed in 100 % of each group. In the CAPS-CA group 100 % can be deemed to have recovered while in the PDS group one case still met criteria according to DSM-IV (50 % recovery rate). This leads to a total recovery rate of 83.3 % in our small sample.

### Treatment modifications and challenges

The treatment aims presented in Table 2 were reached for each patient. However, in the five cases without permanent asylum status the uncertainty about asylum proceedings remained a factor still impairing the feeling of safety in Germany. Table 4 gives an overview of treatment modifications for each patient as reported by the therapist. In one case no modification and, in the other cases, only a few modifications were made. In three cases caregivers were less involved in the treatment, which means a change in TF-CBT protocol. In almost every therapy more time was spent on the affective modulation component and therapists had to utilize further materials like feeling face cards or games teaching feelings. The modifications regarding the trauma narrative were: more than four sessions spent on this component, creating a life-line before starting with the narrative, and using different ways or materials for talking about the trauma (audiotaping/walking around). An important focus was seen in enhancing a feeling of safety (e.g., feeling safe in Germany, distinction between circumstances in home vs. host country). Altogether, therapists reported no difficulties in working according to the TF-CBT manual and modifications were realized without any major changes to the protocol.

**Table 4:** Treatment modifications of each patient

No	Number of treatment sessions	Modification regarding treatment content (PRACTICE), material and setting
1	12 sessions	**A:** terms for emotions were not known in mother tongue; practicing naming feelings, use of face feelings cards and skills box
		**T:** creating a life-line before starting the trauma narrative
		**C:** therapist was more directive in cognitive processing II
2	12 sessions	**P:** psychoeducation about asylum procedure and asylum right in Germany
		**R:** practicing several different relaxation techniques
		**A:** using a feelings game; more time was spent describing and classifying feelings
3	25 sessions	**A:** 6 sessions were spent naming and regulating feelings
		**T:** 12 sessions were spent creating the trauma narrative
		**E:** focus on enhancing a feeling of safety over 4 sessions (What makes me feel safe in Germany? How can I get a perspective in Germany?)
		*Less caregiver involvement (3 sessions). The patient was accompanied by a translator especially at the beginning of therapy and during trauma narrative. He only translated when necessary.*
4	12 sessions	**--**
5	25 sessions	**P:** psychoeducation about dissociation
		**R:** practicing more relaxation techniques over 3 sessions; PMR was practiced in almost every session.
		**A:** feelings were named in both languages; skills for emotion regulation; more time was spent describing and classifying feelings
		**T:** trauma narrative was written in both languages, was created over 15 sessions; to prevent dissociation the patient walked around during the creation of the narrative
		**C:** more time was spent on cognitive processing II
		**E:** enhancing feeling of safety regarding Germany as a safe country
6	28 sessions	**A:** 5 sessions were spent naming and regulating feelings
		**T:** the trauma narrative has been audio-recorded without stopping to translate; it was translated in full afterwards; 16 sessions were spent on creating the trauma narrative.
		**E:** focus on feeling of safety and looking back (4 sessions; e.g. feeling safe in Germany; how would life have been different if there had been peace in Afghanistan; how to deal with uncertainty about whether family is alive).
		*Less caregiver involvement (3 sessions). A translator attended therapy as a companion and the trauma narrative was shared with him.*

*P* psychoeducation, *R* relaxation, *A* affective modulation, *C* cognitive processing II, *T* trauma narrative, *E* enhancing future safety

The therapists’ responses to challenges in the treatment of URMs can be subsumed in three categories: residence status, family, and severe symptomatology. Often, the adolescents’ residence status was not clear so therapists first had to find out more about this in order to understand additional stressful factors and to provide psychoeducation on that topic. On the one hand, the challenges regarding family were concerns about the safety of family members. On the other hand, families in the home countries often had high expectations (e.g., regarding sending money). A further challenge mentioned by two therapists is that the patient viewed caregivers from institutions as father or mother substitutes. This led to disappointment as caregivers could not meet this need. Hence, in therapy and especially in conjoint sessions the distinction between caregiver and parent had to be emphasized. Severe symptomatology was particularly manifested by dissociative symptoms, massive sleeping disorders and nightmares.

Therapists were also asked about their experience in working with translators. In the two cases with translators therapists made good experiences. Translators were particularly helpful during the affective modulation component because therapists could learn and ask for further information about the impact of culture-related issues on emotions.

## Discussion

We tested the feasibility of TF-CBT in a small sample of traumatized URMs, a group of patients which up to now has been clearly undersupplied with mental health support. The participating adolescents reported multiple traumatic experiences and severe PTSS, findings that correlate with research from other European host countries. In addition, stressors such as uncertainty about the permanency of residents’ permits and their family’s safety were obvious in almost all patients.

Our results show a clinically significant symptom reduction at posttest for all cases after receiving TF-CBT. Statistically significant changes were indicated by the Wilcoxon signed rank tests, however the results have to be interpreted carefully due to the very small sample size. Shortly after treatment all but one case were considered recovered on the individual level.

Therapists reported few treatment modifications and hardly any difficulties in comparison to other TF-CBT cases. As URMs, by definition, have no parents, professional caregivers were involved. Whereas TF-CBT is regularly conducted with parents or caregivers in an equal number of sessions, in our study some caregivers were less involved in therapies. This is consistent with a review of Lang, Ford, and Fitzgerald [53] and with the above-mentioned study with young refugees [37]. They back the effectiveness of TF-CBT with low levels of parental involvement. Furthermore, the TF-CBT study in Cambodia [39] was promising without any parent sessions. We observed another modification in the affective modulation component, but not in its core elements. Our findings rather suggest that this component should be expanded in the treatment of URMs as adolescents were not familiar with the concept or naming of feelings triggered by cultural diversities. To overcome this problem, therapists used additional materials. Similar to our therapists’ feedback, Murray and Skavenski [38] report on additional help for the work on affective modulation with children in Zambia and Cambodia. Furthermore, in accordance with their publication, the narrative work was supported with lifelines or required more than the customary four sessions. In vivo mastery was scarcely used within our sample. The latter can be explained by completely different environments regarding traumatization and therapy. Altogether, the modifications mentioned are similar to those reported for the TF-CBT treatment of adolescents with complex trauma [54].

URMs seem to be a group that can be successfully treated for PTSS, even though cultural differences exist, as demonstrated in the treatment applications for TF-CBT [38–41]. Nevertheless, mental healthcare professionals rarely use TF-CBT or other evidence-based trauma-focused therapies with URMs. Some authors prefer multimodal interventions to trauma-focused therapy. However, as demonstrated in our case series, trauma-focused psychotherapy adds significantly to the provision of close-meshed support for URMs in terms of their basic and social needs (e.g., school, health care, legal support), support which is usually already provided in industrialized countries like Germany. Also with regard to cost-effectiveness and immediate symptom improvement, short TF-CBT focusing on traumatic stress symptoms may be sufficient for URMs whose basic needs are already met. Even though for our group of URMs TF-CBT seemed highly appropriate, we cannot state any conclusions for the whole population of URMs, consisting of many nationalities, different ages, and diverse cultures.

### Limitations

Several limitations should be considered. First, the results cannot be generalized due to the small sample size and due to the specific expertise and treatment settings in both involved pediatric trauma clinics. In addition, six cases can hardly represent the heterogeneity of the group of URMs. Given the pilot nature of our study, we did not include a control group. Hence, our results do not permit any conclusions about comparisons to spontaneous improvement or alternative treatments. It cannot be ruled out that simply the fact of receiving more attention and having regular appointments with a professional could be sufficient to reduce symptoms in our patient group. Second, we examined a healthcare utilization sample with several differences in terms of number of sessions, involvement of caregivers and assessment (e.g., different instruments, some assessors were blind to study group assignment, others were the therapists themselves). Finally, since we do not report any follow-up data, the long-term treatment success remains to be seen. Especially in this group attaining the age of majority seems to be a risk factor for inter alia depressive symptoms due to the risk of deportation.

## Conclusions

Our results are promising: TF-CBT is applicable and feasible for URMs with severe PTSS. Our therapists’ reports may encourage other clinicians to treat this group of minors, even in cases where an interpreter is needed. Future research should focus on controlled trials with follow-up assessment to permit statements on treatment efficacy and generalizability of results.

## Abbreviations

CAPS-CA: Clinician administered PTSD scale for children and adolescents
CYW: Child and youth welfare
CBT: Cognitive behavioral therapy
DSM-IV: Diagnostic and statistical manual of mental disorders, 4^th^ edition
KIDNET: Narrative exposure therapy for children
NET: Narrative exposure therapy
PDS: Posttraumatic diagnostic scale
PTSD: Posttraumatic stress disorder
PTSS: Posttraumatic stress symptoms
RCT: Randomized controlled trial
TF-CBT: Trauma-focused cognitive behavioral therapy
URMs: Unaccompanied refugee minors

## References

[1] United Nations High Commissioner for Refugees: Asylum Trends 2013. Levels and trends in industrialized countries. 2014. http://unhcr.org/trends2013/. Accessed 27 Nov 2014.

[2] United Nations High Commissioner for Refugees: Refugee children. Guidelines on protection & care. Geneva: UNHCR: 1994.

[3] Ehntholt KA, Yule W (2006). Practitioner review: assessment and treatment of refugee children and adolescents who have experienced war-related trauma. J Child Psychol Psyc.

[4] Lustig SL, Kia-Keating M, Grant-Knight W, Geltman P, Ellis H, Birman D (2004). Review of child and adolescent refugee mental health. J Am Acad Child Adolesc Psychiatry.

[5] Hodes M, Jagdev D, Chandra N, Cunniff A (2008). Risk and resilience for psychological distress amongst unaccompanied asylum seeking adolescents. J Child Psychol Psyc.

[6] Thomas S, Nafees B, Bhugra D (2004). ‘I was running away from death’- the pre-flight experiences of unaccompanied asylum seeking children in the UK. Child Care Hlth Dev.

[7] Vervliet M, Lammertyn J, Broekaert E, Derluyn I (2014). Longitudinal follow-up of the mental health of unaccompanied refugee minors. Eur Child Adoles Psy.

[8] Kinzie JD, Sack WH, Angell RH, Manson SM, Rath B (1986). The psychiatric effects of massive trauma on Cambodian children: I. The children. J Am Acad Child Adolesc Psychiatry.

[9] Papageorgiou V, Frangou-Garunovic A, Iordanidou R, Yule W, Smith P, Vostanis P (2000). War trauma and psychopathology in Bosnian refugee children. Eur Child Adoles Psy.

[10] Servan-Schreiber D, Le Lin B, Birmaher B (1998). Prevalence of posttraumatic stress disorder and major depressive disorder in Tibetan refugee children. J Am Acad Child Adolesc Psychiatry.

[11] Fazel M, Wheeler J, Danesh J (2005). Prevalence of serious mental disorder in 7000 refugees resettled in western countries: a systematic review. Lancet.

[12] Ruf M, Schauer M, Elbert T (2010). Prävalenz von traumatischen Stresserfahrungen und seelischen Erkrankungen bei in Deutschland lebenden Kindern von Asylbewerbern (translation of title: Prevalence of traumatic stress and mental health problems in children of asylum seekers in Germany). Z Klin Psychol-Forsc.

[13] Smid GE, Lensvelt-Mulders GJLM, Knipscheer JW, Gersons BPR, Kleber RJ (2011). Late-onset PTSD in unaccompanied refugee minors: exploring the predictive utility of depression and anxiety symptoms. J Clin Child Adolesc.

[14] Almqvist K, Brandell-Forsberg M (1997). Refugee children in Sweden: post-traumatic stress disorder in Iranian preschool children exposed to organized violence. Child Abuse Negl.

[15] Mollica RF, McInnes K, Poole C, Tor S (1998). Dose-effect relationships of trauma to symptoms of depression and post-traumatic stress disorder among Cambodian survivors of mass violence. Br J Psychiatry.

[16] Mollica RF, Poole C, Son L, Murray CC, Tor S (1997). Effects of war trauma on Cambodian refugee adolescents’ functional health and mental health status. J Am Acad Child Adolesc Psychiatry.

[17] Bean T, Derluyn I, Eurelings-Bontekoe E, Broekaert E, Spinhoven P (2007). Comparing psychological distress, traumatic stress reactions, and experiences of unaccompanied refugee minors with experiences of adolescents accompanied by parents. J Nerv Ment Dis.

[18] Derluyn I, Mels C, Broekaert E (2009). Mental health problems in separated refugee adolescents. J Adolescent Health.

[19] Michelson D, Sclare I (2009). Psychological needs, service utilization and provision of care in a specialist mental health clinic for young refugees: A comparative study. Clin Child Psychol Psychiatry.

[20] Wiese EBP, Burhorst I (2007). The mental health of asylum-seeking and refugee children and adolescents attending a clinic in the Netherlands. Transcult Psychiatry.

[21] Heptinstall E, Sethna V, Taylor E (2004). PTSD and depression in refugee children: associations with pre-migration trauma and post-migration stress. Eur Child Adoles Psy.

[22] Huemer J, Karnik NS, Voelkl-Kernstock S, Granditsch E, Dervic K, Friedrich MH (2009). Mental health issues in unaccompanied refugee minors. Child Adolesc Psychiatry Ment Health.

[23] Thommessen S, Laghi F, Cerrone C, Baiocco R, Todd BK (2013). Internalizing and externalizing symptoms among unaccompanied refugee and Italian adolescents. Child Youth Serv Rev.

[24] Huemer J, Karnik N, Voelkl-Kernstock S, Granditsch E, Plattner B, Friedrich M (2011). Psychopathology in African unaccompanied refugee minors in Austria. Child Psychiat Hum D.

[25] Vervliet M, Demott M, Melinda A, Jakobsen M, Broekaert E, Heir T (2014). The mental health of unaccompanied refugee minors on arrival in the host country. Scand J Psychol.

[26] Bean TM, Eurelings-Bontekoe E, Spinhoven P (2007). Course and predictors of mental health of unaccompanied refugee minors in the Netherlands: One year follow-up. Soc Sci Med.

[27] Jensen TK, Skårdalsmo EMB, Fjermestad KW (2014). Development of mental health problems − a follow-up study of unaccompanied refugee minors. Child Adolesc Psychiatry Ment Health.

[28] Bean T, Eurelings-Bontekoe E, Mooijaart A, Spinhoven P (2006). Factors associated with mental health service need and utilization among unaccompanied refugee adolescents. Adm Policy Ment Hlth.

[29] Cohen JA, Mannarino AP, Deblinger E (2006). Treating trauma and traumatic grief in children and adolescents.

[30] Leenarts LEW, Diehle J, Doreleijers TAH, Jansma EP, Lindauer RJL (2013). Evidence-based treatments for children with trauma-related psychopathology as a result of childhood maltreatment: a systematic review. Eur Child Adoles Psy.

[31] Tyrer RA, Fazel M (2014). School and community-based interventions for refugee and asylum seeking children: a systematic review. PLoS One.

[32] Eberle-Sejari R, Nocon A, Rosner R (2015). Zur Wirksamkeit von psychotherapeutischen Interventionen bei jungen Flüchtlingen und Binnenvertriebenen mit posttraumatischen Symptomen – ein systematischer Review (translation of title: Efficacy of psychotherapeutic interventions for young refugees and internally displaced persons – a systematic review). Kindh Entwickl.

[33] Cary CE, McMillen JC (2012). The data behind the dissemination: a systematic review of trauma-focused cognitive behavioral therapy for use with children and youth. Child Youth Serv Rev.

[34] Diehle J, Opmeer BC, Boer F, Mannarino AP, Lindauer RJL (2015). Trauma-focused cognitive behavioral therapy or eye movement desensitization and reprocessing: what works in children with posttraumatic stress symptoms? A randomized controlled trial. Eur Child Adoles Psy.

[35] Jensen TK, Holt T, Ormhaug SM, Egeland K, Granly L, Hoaas LC (2014). A randomized effectiveness study comparing trauma-focused cognitive behavioral therapy with therapy as usual for youth. J Clin Child Adolesc.

[36] Murray LK, Cohen JA, Ellis BH, Mannarino A (2008). Cognitive behavioral therapy for symptoms of trauma and traumatic grief in refugee youth. Child Adol Psych Cl.

[37] Schottelkorb AA, Doumas DM, Garcia R (2012). Treatment for childhood refugee trauma: a randomized controlled trial. Int J Play Ther.

[38] Murray LK, Skavenski SA, Cohen JA, Mannarino AP, Deblinger E (2012). International settings. Trauma-focused CBT for children and adolescents: treatment applications.

[39] Bass J, Bearup L, Bolton P, Murray L, Skavenski S (2011). Implementing trauma-focused cognitive behavioral therapy (TF-CBT) among formerly trafficked-sexually abused girls in Cambodia: a feasibility study.

[40] Murray LK, Familiar I, Skavenski S, Jere E, Cohen J, Imasiku M (2013). An evaluation of trauma focused cognitive behavioral therapy for children in Zambia. Child Abuse Negl.

[41] Murray LK, Dorsey S, Skavenski S, Kasoma M, Imasiku M, Bolton P (2013). Identification, modification, and implementation of an evidence-based psychotherapy for children in a low-income country: the use of TF-CBT in Zambia. Int J Ment Health Syst.

[42] Kliethermes M, Wamser R, Cohen JA, Mannarino AP, Deblinger E (2012). Adolescents with complex trauma. Trauma-focused CBT for children and adolescents: treatment applications.

[43] University of Ulm, Federal Ministry of Education and Research. Effectiveness of trauma-focused cognitive-behavioral therapy for children with post-traumatic stress disorder (TreatChildTrauma; TCT). https://clinicaltrials.gov/ct2/show/NCT01516827 (2011). Accessed 27 Nov 2014.

[44] Nader KO, Kriegler JA, Blake DD, Pynoos RS (1994). Clinical Administered PTSD Scale, Child and Adolescent Version (CAPS-CA).

[45] Foa EB, Cashman L, Jaycox L, Perry K (1997). The validation of a self-report measure of posttraumatic stress disorder: the posttraumatic diagnostic scale. Psychol Assessment.

[46] Kaufman J, Birmaher B, Brent D, Rao U, Flynn C, Moreci P (1997). Schedule for Affective Disorders and Schizophrenia for School-Age Children-Present and Lifetime Version (K-SADS-PL): initial reliability and validity data. J Am Acad Child Adolesc Psychiatry.

[47] Wechsler D (2004). The Wechsler intelligence scale for children.

[48] Steil R, Füchsel G (2006). Interviews zu Belastungsstörungen bei Kindern und Jugendlichen.

[49] Griesel D, Wessa M, Flor H (2006). Psychometric qualities of the German version of the posttraumatic diagnostic scale (PDS). Psychol Assessment.

[50] Jacobson NS, Truax P (1991). Clinical significance: a statistical approach to defining meaningful change in psychotherapy research. J Consult Clin Psych.

[51] Matulis S, Resick PA, Rosner R, Steil R (2014). Developmentally adapted cognitive processing therapy for adolescents suffering from posttraumatic stress disorder after childhood sexual or physical abuse: A pilot study. Clin Child Fam Psych.

[52] Ogles BO, Lambert M (2013). Measuring change in psychotherapy research. Handbook of psychotherapy research and behavior change.

[53] Lang JM, Ford JD, Fitzgerald MM (2010). An algorithm for determining use of trauma-focused cognitive-behavioral therapy. Psychother-Theor Res.

[54] Cohen JA, Mannarino AP, Kliethermes M, Murray LA (2012). Trauma-focused CBT for youth with complex trauma. Child Abuse Negl.

